# Multisystem inflammatory syndrome in children (MIS-C) and sepsis differentiation by a clinical and analytical score: MISSEP score

**DOI:** 10.1007/s00431-023-05168-w

**Published:** 2023-09-07

**Authors:** María Hernández-García, Elies Roldan-Berengue, Carmina Guitart, Mònica Girona-Alarcón, Guillermo Argüello, Rosa Pino, Mariona F. de Sevilla, Juan José García-García, Iolanda Jordan

**Affiliations:** 1 Paediatrics Department, Hospital Sant Joan de Déu Barcelona, Barcelona, Spain; 2 Infectious Diseases and Microbiome, Institut de Recerca Sant Joan de Déu (IRSJD), Barcelona, Spain; 3 Department of Medicine, Universitat Internacional de Catalunya, Barcelona, Spain; 4 Paediatric Intensive Care Unit, Hospital Sant Joan de Déu Barcelona, Barcelona, Spain; 5 Faculty of Computer Science, Multimedia and Telecommunications, Universitat Oberta de Catalunya, Barcelona, Spain; 6 Statistics and Operations Research, Universidad de Oviedo, Oviedo, Asturias Spain; 7 Faculty of Medicine and Health Sciences, Universitat de Barcelona, Barcelona, Spain; 8 Centro de Investigación Biomédica en Red de Epidemiología y Salud Pública (CIBER-ESP), Instituto de Salud Carlos III, Madrid, Spain

**Keywords:** MIS-C, Sepsis, COVID-19, SARS-CoV-2, Biomarkers, Diagnostic score

## Abstract

Differential diagnosis between Multisystem Inflammatory Syndrome in Children (MIS-C) and other causes of systemic inflammatory response such as sepsis is complex. The aims were to evaluate the differences between pediatric patients with MIS-C and sepsis and to develop a score to distinguish both entities. This was a retrospective study that compared demographic, clinical, diagnostic, and therapeutic data of pediatric patients with MIS-C (cohort 2020–2022) and sepsis (cohorts 2010–2014 and 2017–2018) admitted to a Pediatric Intensive Care Unit (PICU) of a tertiary care hospital. A diagnostic score was developed with variables that differentiated the two conditions. Twenty-nine patients with MIS-C were identified, who were matched 1:3 with patients with sepsis (*n* = 87). Patients with MIS-C were older (10 vs. 4 years old), and the majority were male (69%). Clinical characteristics that demonstrated differences were prolonged fever and signs and symptoms affecting skin-mucosa and gastrointestinal system. Leukocytes, PCT, and ferritin were higher in sepsis, while thrombocytopenia, lymphopenia, and elevated fibrinogen and adrenomedullin (biomarker with a role for the detection of invasive infections) were more frequent in MIS-C. MIS-C patients presented greater myocardial dysfunction (*p* < 0.001). Five criteria were selected and included in the MISSEP score after fitting them into a multivariate logistic regression model: fever > 48 hours (20 points), thrombocytopenia < 150 × 10^3^/µL (6 points), abdominal pain (15 points), conjunctival erythema (11 points), and Vasoactive Inotropic Score (VIS) > 10 (7 points). The cutoff > 25 points allowed to discriminate MIS-C from sepsis with a sensitivity of 0.89 and specificity of 0.95.

*     Conclusion*: MIS-C phenotype overlaps with sepsis. MISSEP score could be useful to distinguish between both entities and direct specific treatment.

**Table:** 

**What is Known:** *• Differential diagnosis between Multisystem Inflammatory Syndrome in Children (MIS-C) and other causes of systemic inflammatory response such as sepsis is complex.* *• It is essential to establish an accurate initial diagnosis and early specific treatment in both cases of MIS-C and sepsis to improve the prognosis of these patients.*	
**What is New:** *• Patients with MIS-C are older and have characteristic symptoms of prolonged fever, gastrointestinal symptoms, skin-mucosal involvement, and greater myocardial dysfunction, compared to patients with sepsis.* *• The use of diagnostic scores, such as the MISSEP score, can be very useful to distinguish between the two entities and help direct specific treatment.*

**What is Known:**

*• Differential diagnosis between Multisystem Inflammatory Syndrome in Children (MIS-C) and other causes of systemic inflammatory response such as sepsis is complex.*

*• It is essential to establish an accurate initial diagnosis and early specific treatment in both cases of MIS-C and sepsis to improve the prognosis of these patients.*

**What is New:**

*• Patients with MIS-C are older and have characteristic symptoms of prolonged fever, gastrointestinal symptoms, skin-mucosal involvement, and greater myocardial dysfunction, compared to patients with sepsis.*

*• The use of diagnostic scores, such as the MISSEP score, can be very useful to distinguish between the two entities and help direct specific treatment.*

## Introduction

Multisystem Inflammatory Syndrome in Children (MIS-C) is a post-infection complication occurring between 2 and 6 weeks after SARS-CoV-2 infection [1–4]. Although uncommon, being described as only appearing in < 1% of children with confirmed SARS-CoV-2 infection, it can lead to admission to Pediatric Intensive Care Units (PICU) given its quick onset and worsening if not properly managed [1]. The clinical phenotype of MIS-C overlaps with other inflammatory conditions such as Kawasaki Disease, Sepsis, and Staphylococcal or treptococcal Toxic Shock Syndrome (STSS), which complicates differential diagnosis specially during the early stages of the disease, when establishing specific and early treatment is crucial [5–8].

Sepsis, one of those conditions which can mimic the manifestations of MIS-C, is a life-threatening organ dysfunction caused by a dysregulated host response to infection [9, 10]. It is a leading cause of morbidity and mortality in children worldwide, with an estimated incidence of 1.2 million cases per year [11]. Sepsis, as well as MIS-C, also presents with fever, elevated inflammatory biomarkers, hypotension or shock, coagulopathy, and multiorgan dysfunction [9]. The similarity of the clinical characteristics of both entities has led to a diagnostic challenge for physicians during this pandemic [12].

Moreover, it must be considered that MIS-C is a diagnosis of exclusion, which includes ruling out other bacterial causes of inflammation, such as sepsis. For this reason, all patients are initially treated with antibiotics as if for sepsis and, if MIS-C is suspected, they are given supportive therapies with intravenous immunoglobulin (IVIG) and intravenous methylprednisolone [13, 14].

Given the importance of establishing an accurate initial diagnosis and specific treatment in both MIS-C and sepsis cases, this retrospective study was conducted. The aims were to compare their epidemiological, clinical, diagnostic, and therapeutic characteristics and to design a diagnostic score that helps to differentiate them and thus be able to initiate targeted treatment as soon as possible to improve their prognosis.

## Materials and methods

This was a retrospective study that compared epidemiological, clinical, diagnostic, and therapeutic data of pediatric patients (< 19 years old) diagnosed with sepsis and MIS-C, admitted to the PICU of Sant Joan de Déu Hospital. This is a tertiary care level pediatric hospital with 345 beds (28 PICU beds) and estimated to manage the 20% of all annual hospitalizations in Catalonia (Spain). The objectives of the study were to compare the characteristics of patients with MIS-C and patients with sepsis and to design a diagnostic score that helps to differentiate both entities.

Our total sample consisted of 116 patients: from 29 patients with MIS-C we selected 87 patients with sepsis, resulting in a ratio of MIS-C to sepsis of 1:3 (since we only had 29 examples of MIS-C patients, we chose a ratio of 1:3 in order to have a sufficiently balanced data set with an adequate number of observations to fit a predictive model). The ratio of female to male in the MIS-C cohort was 1:2.2, so this ratio was replicated for the sepsis cohort. Pairing by age was tried but not achieved because the median age for MIS-C patients was greater.

The primary outcomes of the study were hours of fever at admission and cardiac dysfunction. These were selected based on their clinical importance for both sepsis and MIS-C according to previous studies. In addition, other secondary outcomes were also determined: epidemiological (age, gender, comorbidities, stay, outcome), clinical (signs and symptoms), diagnostic (other laboratory data at admission, echocardiographic cardiac dysfunction data), and therapeutic (requirement of inotropic support and/or fluid therapy).

MIS-C patients were diagnosed following the World Health Organization (WHO) classification criteria [15], which implies children and adolescents 0–19 years old, with prolonged fever (> 3 days), elevated inflammatory biomarkers and two of the following: rash, conjunctivitis or muco-cutaneous inflammation signs, hypotension or shock, cardiac dysfunction, coagulopathy, or gastrointestinal symptoms. The definition also includes the absence of other bacterial causes of inflammation (such as sepsis) as well as the evidence of previous SARS-CoV-2 infection (by RT-PCR, antigen test, or positive serology) or the history of contact with a COVID-19 patient. In the MIS-C cohort, exclusion criteria were being hospitalized but not admitted to the PICU and not requiring fluid therapy or inotropic support.

Sepsis patients were diagnosed based on International Pediatric Sepsis Consensus definition [9]. Sepsis is defined as a Systemic Inflammatory Response Syndrome (SIRS) as a result of suspected or proven infection. Severe sepsis is defined as a sepsis with cardiovascular organ dysfunction, acute respiratory distress syndrome, or two or more other organ dysfunctions. In the sepsis cohort, the exclusion criterion was having history of immunosuppression secondary to pathologies such as leukemia or immunosuppressive treatments like chemotherapy (given the alteration in laboratory data secondary to these conditions).

MIS-C patients were attended and prospectively recorded between 2020 and beginnings of 2022. All included sepsis patients had been admitted before the COVID-19 pandemic (2010–2014 and 2017–2018 cohorts) and had been included in a prospective database for different studies [16–18]. Patients with sepsis from the years 2020–2022 were not included because there was not enough sample in those years and also precisely to avoid confounding factors with the MIS-C group.

Regarding MIS-C patients, it was reported whether or not they had been vaccinated against SARS-CoV-2 before admission. However, it was not possible to differentiate whether the positive IgGs were secondary to natural infection or vaccination.

All the data of interest were extracted from electronic records entered by the pediatric critical care physicians who had attended each of the patients. No personally identifiable data was collected during the conduct of this study. Unfortunately, being a retrospective study, there was information on clinical or laboratory variables that was not collected in all patients.

Written informed consent was obtained from all patients or their legal guardians. The study was done in accordance with the Helsinki declaration and approved by the Sant Joan de Déu Ethical Assistant Committee (PIC-180–2).

### Statistical analysis

Categorical variables were expressed as proportions while continuous variables were expressed as median and interquartile range (IQR). The comparison of categorical variables was performed using the Chi-Square test and Fisher’s exact test; continuous variables were compared using the Student *t*-test. All tests were two-sided, and *p* values less than 0.05 were considered to indicate a statistically significant difference. Statistical analyses were done using SPSS, version 25.0.

One of our main objectives was to develop a diagnostic prediction model that could help distinguish patients with MIS-C and sepsis. Although the training data was manually built and there were not many missing values, we took missing values for categorical variables to mean the absence of the variable and imputed missing values in continuous variables with their class mean value. Before training the model, we applied the Boruta feature selection algorithm [19] to select the most important predictors. Subsequently, with these variables, we trained a logistic regression, scaling the resulting regression coefficients, multiplying them by 100, and rounding to the nearest integer. The sum of all the scaled coefficients constituted a score that was our final outcome. Binary variables included in the model were coded as present or absent. Threshold selection was based on a ROC curve analysis, selecting the value at which sensitivity plus specificity were maximized. To overcome the limited amount of data, we used a tenfold cross-validation to validate model performance as recommended in [20] and l2-penalty to avoid overfitting [21]. We used precision, sensitivity (recall), and f1-score metrics to measure model performance, calculated as follows:

$$precision=\frac{TP}{TP+FP}$$

$$sensitivity=\frac{TP}{TP+FN}$$

$$f1-score=\frac{2*precision*sensitivity}{precision+sensitivity}$$

## Results

Our total sample consisted of 116 patients: 29 patients with MIS-C and 87 patients with sepsis (ratio 1:3). The global median age was 5.5 years old (IQR 2.1–11.3), and most of them were male (69%). MIS-C patients were older (median 10 years; IQR 6.9–13.8) than sepsis patients (median 4 years; IQR 1.6–8.5). Sepsis patients had more frequently underlying medical conditions (31% vs. 7%; *p* = 0.009). In comparison to sepsis patients, patients with MIS-C had shorter median hospital stay (8 vs. 13 days; *p* = 0.007) and shorter PICU stay (3 vs. 6 days; *p* = 0.008). Five patients died, all of them belonged to the sepsis group.

For patients diagnosed with MIS-C, the evidence of previous SARS-CoV-2 infection was documented. Of the 29 MIS-C patients, 29 (100%) had positive IgG serology (4 of them (14%) had been vaccinated against SARS-CoV-2 before admission), 10 (35%) had positive IgM serology, and just 5 out of 22 (23%) still had a positive SARS-CoV-2 PCR on admission.

### Clinical data comparison

In terms of clinical variables, there was a considerable overlap between the signs and symptoms of both entities. Fever was the most frequent symptom; although it was present in 100% of MIS-C cases vs. 81% of sepsis cases (*p* = 0.006), its duration on admission was notably shorter in sepsis cases (median 20 h vs. 96 h; *p* < 0.001). Patients with MIS-C were more likely to have gastrointestinal symptoms such as abdominal pain (83% vs. 15%; *p* < 0.001) and diarrhea (55% vs. 17%; *p* < 0.001). The signs and symptoms that share similarity with Kawasaki disease were clearly more frequent in patients with MIS-C: rash (48% vs. 24%; *p* = 0.01), conjunctival erythema (45% vs. 1%; *p* < 0.001), and muco-cutaneous inflammation such as odynophagia (28% vs. 2%; *p* < 0.001) and oral ulcers (31% vs. 6%; *p* = 0.001). All the epidemiological and clinical data of the patients are described in Table 1.

**Table 1:** Epidemiological and clinical data of interest comparing patients with sepsis and MIS-C

	**Total (*n* = 116)**	**Sepsis (*n* = 87)**	**MIS-C (*n* = 29)**	***p* value**
Epidemiological data				
Age (years old); median (IQR)	5.5 (2.1–11.3)	4 (1.6–8.5)	10 (6.9–13.8)	**<0.001**
Sex (males); *n* (%)	80 (69%)	60 (69%)	20 (69%)	1
Underlying medical condition; *n* (%)	29 (25%)	27 (31%)	2 (7%)	**0.009**
Evolution				
Hospital stay (days); median (IQR)	10.5 (8–17)	13 (8–20)	8 (5.5–10)	**0.007**
PICU stay (days); median (IQR)	4 (2–8)	6 (2–10)	3 (2–5)	**0.008**
Outcome (died); *n* (%)	5 (4%)	5 (6%)	0	0.32
PRISM III score; median (IQR)	5 (2–10.75)	7 (2–12.5)	4 (2–8)	**0.04**
Clinical data				
General clinical data				
Fever; *n* (%)	99 (85%)	70 (81%)	29 (100%)	**0.006**
Hours of fever at admission; median (IQR)	24 (16–48)	20 (12–30)	96 (72–144)	**<0.001**
Bad general condition; *n* (%)	54 (47%)	44 (51%)	10 (35%)	0.13
Asthenia; *n* (%)	15 (13%)	8 (9%)	7 (24%)	0.05
Myalgias; *n* (%)	7 (6%)	1 (1%)	6 (21%)	**0.001**
Cervical adenopathy; *n* (%)	5 (4%)	2 (2%)	3 (10%)	0.09
Gastrointestinal data				
Abdominal pain; *n* (%)	37 (32%)	13 (15%)	24 (83%)	**<0.001**
Diarrhea; *n* (%)	31 (27%)	15 (17%)	16 (55%)	**<0.001**
Vomiting; *n* (%)	50 (43%)	34 (39%)	16 (55%)	0.13
Anorexia; *n* (%)	17 (15%)	12 (14%)	5 (17%)	0.76
Constipation; *n* (%)	4 (3%)	1 (1%)	3 (10%)	**0.04**
Respiratory data				
Cough; *n* (%)	43 (37%)	34 (39%)	9 (31%)	0.43
Rhinorrhea; *n* (%)	37 (32%)	34 (39%)	3 (10%)	**0.004**
Dyspnea; *n* (%)	41 (35%)	32 (37%)	9 (31%)	0.57
Dermatological/muco-cutaneous data				
Rash; *n* (%)	35 (30%)	21 (24%)	14 (48%)	**0.01**
Odynophagia; *n* (%)	10 (9%)	2 (2%)	8 (28%)	**<0.001**
Oral ulcers; *n* (%)	14 (12%)	5 (6%)	9 (31%)	**0.001**
Limb edema; *n* (%)	13 (11%)	9 (10%)	4 (14%)	0.73
Conjunctival erythema; *n* (%)	14 (12%)	1 (1%)	13 (45%)	**<0.001**
Cardiovascular data				
Tachycardia; *n* (%)	90 (78%)	67 (77%)	23 (79%)	0.79
Hypotension; *n* (%)	82 (71%)	57 (66%)	25 (86%)	**0.03**
Oliguria; *n* (%)	26 (22%)	16 (18%)	10 (35%)	0.07
Neurologic data				
Altered level of consciousness; *n* (%)	27 (23%)	25 (29%)	2 (7%)	**0.01**
Headache; *n* (%)	18 (16%)	10 (12%)	8 (28%)	0.07
Irritability; *n* (%)	11 (10%)	10 (12%)	1 (3%)	0.28

All the data indicated in bold are values of *p* < 0.05 that are considered statistically significant

*PICU* pediatric intensive care unit, *PRISM III score* pediatric risk of mortality score III

### Analytical data comparison

Compared with patients with MIS-C, patients with sepsis had a higher leukocyte count (12,800/mm^3^ vs. 9600/mm^3^; *p* = 0.01), whereas thrombocytopenia (119 × 10^3^/µL vs. 225 × 10^3^/µL; *p* = 0.001) and lymphopenia (800/mm^3^ vs. 1600/mm^3^; *p* = 0.02) were more common in patients with MIS-C. In terms of inflammatory biomarkers, procalcitonin (17.5 ng/mL vs. 8.4 ng/mL; *p* = 0.01) and ferritin (1096 µg/L vs. 944 µg/L; *p* = 0.001) were higher in sepsis, while fibrinogen (6.4 g/L vs. 4.6 g/L; *p* = 0.001) and adrenomedullin (1.72 nmol/L vs. 1.47 nmol/L; *p* = 0.01) were higher in MIS-C. Regarding cardiovascular affection, despite the fact that no significant differences were found in cardiac biomarkers, it was observed that patients with MIS-C presented more cardiac dysfunction on echocardiography (left ventricular ejection fraction, LVEF < 50%: 48% vs. 13%; *p* < 0.001). All the diagnostic and therapeutic data are described in Table 2.

**Table 2:** Diagnostic and therapeutic data of interest comparing patients with sepsis and MIS-C

	**Total (*n* = 116)**	**Sepsis (*n* = 87)**	**MIS-C (*n* = 29)**	***p* value**
Laboratory data (at admission)				
Hemogram parameters				
Leukocyte count (/mm^3^); median (IQR)	12,500 (6200–18,200)	12,800 (6900–21,725)	9600 (5600–13,350)	**0.01**
Lymphocyte count (/mm^3^); median (IQR)	1200 (600–2500)	1600 (725–2754)	800 (400–1250)	**0.02**
Neutrophil count (/mm^3^); median (IQR)	7200 (3600–13,700)	7300 (3075–14,075)	6700 (4000–11,700)	0.97
Platelet count (× 10^3^/µL); median (IQR)	194 (113–305)	225 (157–351)	119 (100–178)	**0.001**
Coagulation parameters				
Prothrombin time (%); median (IQR)	60.2 (47.5–72.5)	58.7 (45.5–71.3)	64.7 (51.7–80.25)	0.20
Partial thromboplastin time (seconds); median (IQR)	30.2 (26.5–34.8)	30.3 (27.4–36)	28.5 (24.9–33.1)	0.06
Fibrinogen (g/L); median (IQR)	5.1 (4.45–6.45)	4.6 (4.05–5.85)	6.4 (4.9–7.2)	**0.001**
D-dimer (mg/L); median (IQR)	**	**	6.25 (3.29–8.14)	**
Biochemical parameters				
Glucose (mg/dL); median (IQR)	101 (84–131)	98.5 (80.5–121)	137 (122–178)	**0.02**
Creatinine (mg/dL); median (IQR)	0.58 (0.44–0.75)	0.53 (0.39–0.75)	0.63 (0.56–0.75)	0.35
Urea (mg/dL); median (IQR)	26.3 (19–42.9)	26 (17–43.8)	26.9 (21.9–42.5)	0.63
ALT (UI/L); median (IQR)	22 (15–48)	20.5 (12–42.5)	40 (20–51.5)	0.81
AST (UI/L); median (IQR)	33 (24–52)	30 (18–52.7)	39 (28.5–52.5)	0.57
Total bilirubin (mg/dL); median (IQR)	11.6 (9.1–19.7)	12.7 (9.5–19.9)	9.8 (7.3–19.2)	0.83
Conjugated bilirubin (mg/dL); median (IQR)	7.1 (5.3–9.6)	7.3 (4.1–8.2)	7.1 (5.4–11.2)	0.56
Protein (g/L); median (IQR)	56 (51–64)	55 (46–64.5)	58 (53–62.5)	0.83
Albumin (g/L); median (IQR)	26 (22–30.3)	26.5 (23–31.8)	25.5 (20.8–29)	0.35
ESR (mm); median (IQR)	13 (4.8–30)	7 (3.3–26.5)	18 (6.5–31.8)	0.19
C-reactive protein (mg/L); median (IQR)	136.8 (51.2–244)	87 (39.3–215.3)	230.9 (156.2–295.3)	0.41
Procalcitonin (ng/mL); median (IQR)	11.7 (2–43.8)	17.5 (1.7–49.8)	8.4 (2.4–16.9)	**0.01**
Lactate (mmol/L); median (IQR)	2.15 (1.48–3.2)	1.9 (1.3–3.08)	2.75 (1.78–3.33)	0.38
Adrenomedullin (nmol/L); median (IQR)	1.53 (0.94–2.82)	1.47 (0.86–3.45)	1.72 (1.26–2.40)	**0.01**
Ferritin (µg/L); median (IQR)	1008.3 (366.2–1748.3)	1096.9 (360.8–3829.8)	944.6 (369.9–1479.1)	**0.001**
IL-6 (pg/mL); median (IQR)	**	**	158 (61–552)	**
Ionogram parameters				
Sodium (mmol/L); median (IQR)	137 (135–140)	137 (135–141)	135 (132–138)	**0.003**
Potassium (mmol/L); median (IQR)	3.8 (3.4–4)	3.8 (3.4–4.1)	3.7 (3.5–4)	0.70
Chlorine (mmol/L); median (IQR)	106 (102–111)	107 (103–111)	102 (99–108)	0.08
Calcium (mmol/L); median (IQR)	1.18 (1.09–1.24)	1.16 (1.07–1.23)	1.19 (1.16–1.27)	**0.01**
Cardiovascular affection (diagnostic and therapeutic)				
Troponin (ng/mL); median (IQR)	0.068 (0.010–0.830)	0.010 (0.000–0.770)	0.082 (0.014–0.906)	0.44
NT-ProBNP (ng/L); median (IQR)	6839 (4114–21,501)	13,169 (3089–23,250)	6839 (4208–19,167)	0.71
Creatine kinase (UI/L); median (IQR)	70.5 (35.5–282.8)	115 (40.5–346)	59 (33–94)	0.30
Cardiac dysfunction (LVEF < 50%); *n* (%)	25/116 (22%)	11/87 (13%)	14/29 (48%)	**<0.001**
Requirement of inotropic support; *n* (%)	72/116 (62%)	53/87 (61%)	19/29 (66%)	0.65
VIS; median (IQR)	12 (8–25)	10 (8.5–26)	15 (8–25)	0.55

All the data indicated in bold are values of *p* < 0.05 that are considered statistically significant

*ALT* alanine aminotransferase, *AST* aspartate aminotransferase, *ESR* erythrocyte sedimentation rate, *IL-6* interleukin-6, *NT-ProBNP* N-terminal pro-brain natriuretic peptide, *LVEF* left ventricular ejection fraction, *VIS* vasoactive inotropic score

**Missing values

### Diagnostic score

We designed a diagnostic score named MISSEP score, after the conjunction of the words MIS-c and SEPsis. Feature selection algorithm resulted in the selection of five variables that distinguished well between both conditions in previous exposed analysis: hours of fever, platelets, abdominal pain (1 if experienced by the patient, 0 otherwise), conjunctival erythema (1 if experienced by the patient, 0 otherwise), and need for vasoactive drugs (measured as Vasoactive Inotropic Score; VIS). We fitted a multivariate logistic regression model using these five variables and then scaled the regression coefficients to integer values to build the final score (see Table 3).

**Table 3:** Variables included in the score calculation, beta values of the logistic regression, and the points of each variable for scoring

**Variable**	***β***	***p* value**	**Number of points (criteria for scoring)**
Fever (≤ 48 hours)	2.0067	< 0.001	0 (≤ 48 hours of fever) or 20 (> 48 hours of fever)
Thrombocytopenia (platelets < 150 × 10^3^/µL)	0.574	< 0.001	0 (no) or 6 (yes)
Abdominal pain	1.461	< 0.001	0 (no) or 15 (yes)
Conjunctival erythema	1.057	< 0.001	0 (no) or 11 (yes)
Vasoactive Inotropic Score (VIS)	0.657	< 0.001	0 (VIS ≤ 10) or 7 (VIS > 10)

**Fig. 1:**
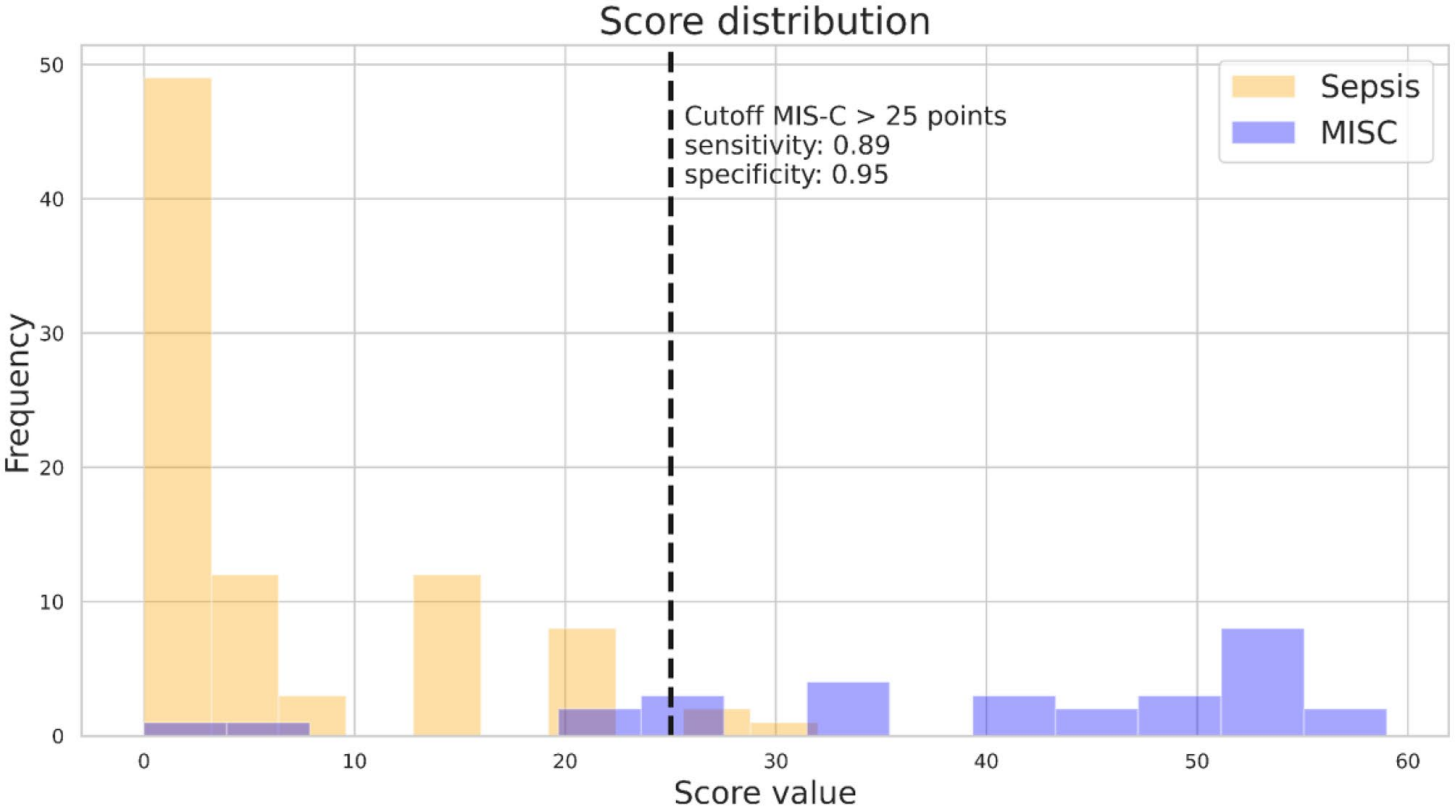
Score distribution of all available patients. Sepsis patients are shown in yellow and MIS-C patients in blue. Dashed line shows the cutoff point. Sensitivity of the model: 0.89 and specificity: 0.95

From a clinical perspective, the score would tell us that MIS-C patients compared to those with sepsis present with more hours of fever, have a lower platelet count, are more likely to have abdominal pain and conjunctival erythema, and have higher VIS values (increased need for inotropic/vasoactive treatment) (see Fig. 1).

We fitted the regression using the whole data set, with a tenfold cross-validation yielding 0.89, 0.86, and 0.88 for precision, sensitivity, and f1-score metrics respectively for MIS-C class and equivalently 0.95, 0.97, and 0.96 for the sepsis class.

## Discussion

Our study assesses the similarities and differences of pediatric patients admitted to the PICU with diagnoses of MIS-C and sepsis. In addition, it provides a diagnostic score: the MISSEP score, named after the conjunction of the words MIS-c and SEPsis. It includes five relevant clinical, laboratory, and therapeutic variables, such as hours of fever, platelets, abdominal pain, conjunctival erythema, and need for vasoactive drugs, whose values have shown differences that make it possible to distinguish between both entities. In order to optimize the management of patients with MIS-C, other authors have proposed diagnostic scores with the aim to be a guide for physicians to help differentiate MIS-C from other conditions [5, 22]. However, as far as we know, to date no score has been designed to help differentiate MIS-C from sepsis. We believe that the MISSEP score distinguishes well between both entities, with high sensitivity and specificity, and that despite being a diagnostic score, secondarily it can also help direct the specific treatment of each of the entities, starting it early and thus improving the prognosis of these patients. From a clinical point of view, the score determines that patients with MIS-C, compared to those with sepsis, present with more hours of fever, have a lower platelet count, and are more likely to have abdominal pain and conjunctival erythema, as well as an increased need for inotropes (measured by the VIS score). Although the score has been validated internally, future external validation with a larger number of patients may improve its application.

The clinical phenotype of MIS-C overlapping with other inflammatory conditions such as COVID-19, Kawasaki disease, and STSS has led to a diagnostic challenge for physicians during this pandemic [5, 6, 8, 22, 23]. The same similarities in the clinical characteristics and laboratory biomarkers have been observed with sepsis, an entity with a higher incidence, and which is related to considerably higher morbidity and mortality in the pediatric population [11, 24]. While COVID-19 and MIS-C have justifiably dominated the attention of the scientific literature for the past 3 years, the incidence of sepsis has continued to outpace the cases of severe SARS-CoV-2-related conditions in children [12]. The importance of knowing how to differentiate MIS-C and sepsis lies in the fact that each one has a different pathophysiology and therefore each one needs a specific treatment.

From an epidemiological point of view, it has been described that sepsis has a higher incidence in patients under 5 years of age, especially in neonates [25]. On the contrary, although it is true that cases of a multisystem inflammatory syndrome associated with SARS-CoV-2 have been described in neonates [26], MIS-C typically occurs in older children, around 5–15 years old [1–3]. In our cohort, patients with MIS-C were characteristically older compared with patients with sepsis. On the other hand, patients with MIS-C do not usually have previous comorbidities; however, in patients with sepsis, as observed in our study, the presence of comorbidities has been described in up to 30–50% of cases [11]. In addition, in relation to the greater severity associated with sepsis, in our cohort we observed that these patients had greater morbidity (longer hospital and PICU stay) and mortality, in comparison to MIS-C patients.

Considering the standardized definitions of MIS-C and sepsis, overlaps have been observed in their diagnostic criteria: the presence of fever, elevated inflammatory biomarkers, hemodynamic dysfunction, and other organ dysfunctions are common in both entities [9, 15]. Fever, the guiding symptom of MIS-C, present in 100% of MIS-C cases in our cohort, usually lasts several days in these patients [1, 3, 27]. However, in cases of sepsis, fever is not a sine qua non condition, in fact, hypothermia can occur, although characteristically when fever is present in septic patients it has a very acute onset, lasting a few hours [9]. In our cohort, there was a statistically significant difference in the duration of fever at admission between both entities, being the duration of fever in cases of sepsis much shorter (20 hours vs. 96 hours).

Both MIS-C and sepsis are associated with hyperinflammation, and both have the potential to develop multi-organ dysfunction. As described in the literature and observed in our study, in MIS-C gastrointestinal symptoms predominate, as well as Kawasaki-like symptoms and signs with skin and mucosa involvement, such as odynophagia, oral ulcers, rash, and conjunctival erythema [1–3, 27].

Regarding biomarkers, characteristic alterations described in the literature to be observed in practically all patients with MIS-C are thrombocytopenia, lymphopenia, and hypoalbuminemia, as well as elevation of C-reactive protein, fibrinogen, D-dimer, and ferritin [28]. Some authors have tried to find patterns of specific inflammatory markers that could distinguish MIS-C from other hyperinflammatory syndromes, such as COVID-19 [29], Kawasaki disease, and macrophage activation syndrome [30]. For instance, Diaz et al. further developed the central role of IL-6, as IL-6 inhibitors prescribed as immunomodulators in cases refractory to standard therapy turned out to be effective. They compared the plasma concentration of IL-6 between critically ill children with MIS-C and sepsis, noting that the concentration of IL-6 rose much more in patients with sepsis than in patients with MIS-C [31]. In our study, in addition to the laboratory abnormalities usually observed in these patients, we compared two other biomarkers: procalcitonin (PCT) and MR-pro adrenomedullin (ADM), two biomarkers that have previously shown utility in the diagnosis of sepsis [17, 32]. PCT is a widely used biomarker in daily clinical practice, having been shown to be specific for acute bacterial infections, and ADM is a relatively new biomarker that has also been proposed as a good tool for the detection of invasive infections. We found that PCT was increased with a statistically significant difference in the sepsis group, compared to the MIS-C group. However, the opposite occurred with ADM, which was statistically significantly higher in the MIS-C group.

As regards with the hemodynamic involvement, in our study we observed that patients with MIS-C had greater myocardial dysfunction at the echocardiographic level (left ventricular ejection fraction; LVEF < 50%), compared to the sepsis group. In addition, patients with MIS-C had a greater need for inotropes, although without showing statistically significant differences. Other authors have demonstrated the usefulness of the early determination of hemodynamic involvement in MIS-C patients, in order to assess starting inotropic/vasoactive support as soon as necessary and, therefore, to improve prognosis [33, 34].

Another key point to note is that the definition of MIS-C includes ruling out other bacterial causes of inflammation, such as sepsis. Given the similarity of patients with MIS-C and sepsis and knowing that the mortality of the latter increases as the time until the start of effective antimicrobial therapy increases, the guidelines recommend that all patients with suspected MIS-C and signs of shock or organ dysfunction should be treated empirically with broad-spectrum intravenous antimicrobial therapy until bacterial sepsis is ruled out [14]. This is a debatable fact, since antibiotics are not necessary in most patients with MIS-C. Some authors have recorded the use of antibiotics in these patients, determining an administration rate of up to 90% [35, 36]. Guidelines, in addition to recommend starting the use of antibiotics, also recommend carrying out a daily evaluation (with clinical and laboratory data) to assess de-escalating or stopping them based on the clinical course, microbiological findings, and the presence of other clear diagnostic criteria in favor of MIS-C [14]. This misuse of antibiotics can lead to an increase in bacterial resistance, a growing global health problem, which is why it is necessary to evaluate the need for antibiotic therapy in all patients with MIS-C.

### Limitations

The present study has several limitations. First, the small sample size may have generated low-quality statistical results. In addition, the fact that the cohorts of patients with sepsis and MIS-C are from different years may have generated biases. Finally, being a retrospective study, there is information on clinical variables that could not be known, as well as laboratory variables, not collected in all patients.

## Conclusions

Patients with MIS-C who require admission to the PICU have many common characteristics with patients with sepsis, an entity with a higher incidence and associated morbidity and mortality. Having tools to distinguish both entities can optimize the management of these patients. In our study, patients with MIS-C were older and had characteristic symptoms of prolonged fever, gastrointestinal symptoms, skin-mucosal involvement, and greater myocardial dysfunction, compared to patients with sepsis. The use of diagnostic scores, such as the MISSEP score, can be very useful to distinguish between the two entities and help direct specific treatment, improving the prognosis of these patients. Future studies with a larger number of patients may improve the application of the MISSEP score.

## Data Availability

The datasets generated during and/or analysed during the current study are not publicly available due to individual privacy could be compromised, but are available from the corresponding author on reasonable request.

## Abbreviations

ADM: MR-pro adrenomedullin
ALT: Alanine aminotransferase
AST: Aspartate aminotransferase
COVID-19: Coronavirus disease 2019
ESR: Erythrocyte sedimentation rate
IL-6: Interleukin-6
LVEF: Left ventricular ejection fraction
MIS-C: Multisystem inflammatory syndrome in children
NT-ProBNP: N-terminal pro-brain natriuretic peptide
PCT: Procalcitonin
PICU: Pediatric intensive care unit
PRISM III score: Pediatric risk of mortality score III
RT-PCR: Reverse transcription-polymerase chain reaction
SARS-CoV-2: Severe acute respiratory syndrome coronavirus 2
SIRS: Systemic inflammatory response syndrome
STSS: Staphylococcal or streptococcal toxic shock syndrome
VIS: Vasoactive inotropic score
WHO: World Health Organization

## References

[1] Hoste L, Van Paemel R, Haerynck F (2021). Multisystem inflammatory syndrome in children related to COVID-19: a systematic review. Eur J Pediatr [Internet].

[2] Lee KH, Li H, Lee MH, Park SJ, Kim JS, Han YJ et al (2022) Clinical characteristics and treatments of multi-system inflammatory syndrome in children: a systematic review. Eur Rev Med Pharmacol Sci [Internet]. 2022 May [cited 2022 Aug 25];26(9):3342–50. 10.26355/eurrev_202205_28754 10.26355/eurrev_202205_28754 35587087

[3] Abrams JY, Godfred-Cato SE, Oster ME, Chow EJ, Koumans EH, Bryant B (2020). Multisystem inflammatory syndrome in children associated with severe acute respiratory syndrome coronavirus 2: a systematic review. J Pediatr [Internet].

[4] Whittaker E, Bamford A, Kenny J, Kaforou M, Jones CE, Shah P (2020). Clinical characteristics of 58 children with a pediatric inflammatory multisystem syndrome temporally associated with SARS-CoV-2. JAMA [Internet].

[5] Godfred-Cato S, Abrams JY, Balachandran N, Jaggi P, Jones K, Rostad CA (2022). Distinguishing multisystem inflammatory syndrome in children from COVID-19, Kawasaki disease and toxic shock syndrome. Pediatric Infectious Disease Journal [Internet].

[6] Sancho-Shimizu V, Brodin P, Cobat A, Biggs CM, Toubiana J, Lucas CL et al (2021) SARS-CoV-2–related MIS-C: a key to the viral and genetic causes of Kawasaki disease? J Exp Med [Internet]. 218(6). 10.1084/jem.20210446 10.1084/jem.20210446 PMC8080850 33904890

[7] Sudeep KC, Awasthi P, Kumar S, Angurana SK, Nallasamy K, Angrup A et al (2022) MIS-C mimickers: a case series of bacterial enteritis and sepsis mistaken as MIS-C. Indian J Pediatr [Internet]. 89(2):206–206. 10.1007/s12098-021-04019-6 10.1007/s12098-021-04019-6 PMC8579170 34757575

[8] Cherqaoui B, Koné-Paut I, Yager H, Le BF, Piram M (2021). Delineating phenotypes of Kawasaki disease and SARS-CoV-2-related inflammatory multisystem syndrome: a French study and literature review. Rheumatology [Internet].

[9] Goldstein B, Giroir B, Randolph A (2005). International pediatric sepsis consensus conference: definitions for sepsis and organ dysfunction in pediatrics*. Pediatric Critical Care Medicine [Internet].

[10] Weiss SL, Peters MJ, Alhazzani W, Agus MSD, Flori HR, Inwald DP (2020). Surviving sepsis campaign international guidelines for the management of septic shock and sepsis-associated organ dysfunction in children. Pediatric Critical Care Medicine [Internet].

[11] Fleischmann-Struzek C, Goldfarb DM, Schlattmann P, Schlapbach LJ, Reinhart K, Kissoon N (2018). The global burden of paediatric and neonatal sepsis: a systematic review. Lancet Respir Med [Internet].

[12] Weiss SL, Peters MJ, Agus MSD, Alhazzani W, Choong K, Flori HR (2020). Perspective of the surviving sepsis campaign on the management of pediatric sepsis in the era of coronavirus disease 2019*. Pediatric Critical Care Medicine [Internet].

[13] Henderson LA, Canna SW, Friedman KG, Gorelik M, Lapidus SK, Bassiri H (2022). American College of Rheumatology clinical guidance for multisystem inflammatory syndrome in children associated with SARS–CoV-2 and hyperinflammation in pediatric COVID-19: version 3. Arthritis & Rheumatology [Internet].

[14] Schlapbach LJ, Andre MC, Grazioli S, Schöbi N, Ritz N, Aebi C (2021). Best practice recommendations for the diagnosis and management of children with pediatric inflammatory multisystem syndrome temporally associated with SARS-CoV-2 (PIMS-TS; multisystem inflammatory syndrome in children, MIS-C) in Switzerland. Front Pediatr [Internet].

[15] World Health Organization. (2020) Multisystem inflammatory syndrome in children and adolescents with COVID-19 scientific brief 15 May 2020 background [Internet]. 2020 [cited 2022 Sep 1]. Available from: https://www.who.int/publications/i/item/multisystem-inflammatory-syndrome-in-children-and-adolescents-with-covid-19

[16] Vila Pérez D, Jordan I, Esteban E, García-Soler P, Murga V, Bonil V (2014). Prognostic factors in pediatric sepsis study, from the Spanish Society of Pediatric Intensive Care. Pediatric Infectious Disease Journal.

[17] Solé-Ribalta A, Bobillo-Pérez S, Valls A, Girona-Alarcón M, Launes C, Cambra FJ (2020). Diagnostic and prognostic value of procalcitonin and mid-regional pro-adrenomedullin in septic paediatric patients. Eur J Pediatr [Internet].

[18] Solé‐Ribalta A, Launes C, Felipe‐Villalobos A, Balaguer M, Luaces C, Garrido R et al (2022) New multivariable prediction model PEdiatric SEpsis recognition and stratification (PESERS score) shows excellent discriminatory capacity. Acta Paediatr [Internet]. [cited 2023 Jan 18];111(6):1209–19. 10.1111/apa.16321 10.1111/apa.16321 35263468

[19] Kursa MB, Rudnicki WR (2010) Feature selection with the Boruta package. J Stat Softw [Internet]. 2010 [cited 2022 Oct 12];36(11). 10.18637/jss.v036.i11

[20] Collins GS, Reitsma JB, Altman DG, Moons K (2015). Transparent reporting of a multivariable prediction model for individual prognosis or diagnosis (TRIPOD): the TRIPOD statement. BMC Med [Internet].

[21] Pekhimenko GG. Penalizied logistic regression for classification [Internet]. Toronto, ON M5S3L1; 2006 [cited 2023 Jan 18]. Available from: https://www.cs.cmu.edu/~gpekhime/Projects/CSC2515/project.pdf

[22] Kostik MM, Bregel LV, Avrusin IS, Dondurei EA, Matyunova AE, Efremova OS (2021). Distinguishing between multisystem inflammatory syndrome, associated with COVID-19 in children and the Kawasaki disease: development of preliminary criteria based on the data of the retrospective multicenter cohort study. Front Pediatr [Internet].

[23] Lin TA, Leo HL (2021). Characteristics and outcomes of US children and adolescents with multisystem inflammatory syndrome in children (MIS-C) compared with severe acute COVID-19. Pediatrics [Internet].

[24] Schlapbach LJ (2019). Paediatric sepsis. Curr Opin Infect Dis [Internet].

[25] Rudd KE, Johnson SC, Agesa KM, Shackelford KA, Tsoi D, Kievlan DR (2020). Global, regional, and national sepsis incidence and mortality, 1990–2017: analysis for the Global Burden of Disease Study. The Lancet [Internet].

[26] More K, Aiyer S, Goti A, Parikh M, Sheikh S, Patel G (2022). Multisystem inflammatory syndrome in neonates (MIS-N) associated with SARS-CoV2 infection: a case series. Eur J Pediatr [Internet].

[27] Santos MO, Gonçalves LC, Silva PAN, Moreira ALE, Ito CRM, Peixoto FAO et al (2022) Multisystem inflammatory syndrome (MIS-C): a systematic review and meta-analysis of clinical characteristics, treatment, and outcomes. J Pediatr (Rio J) [Internet]. 2022 Jul 1;98(4):338–49. 10.1016/j.jped.2021.08.006 10.1016/j.jped.2021.08.006 PMC9432310 34863701

[28] The Royal College of Paediatrics and Child Health (2020) Guidance: paediatric multisystem inflammatory syndrome temporally associated with COVID-19 [Internet]. 2020 May [cited 2022 Sep 1]. Available from: https://www.rcpch.ac.uk/resources/paediatric-multisystem-inflammatory-syndrome-temporally-associated-covid-19-pims-guidance

[29] Zhao Y, Yin L, Patel J, Tang L, Huang Y (2021). The inflammatory markers of multisystem inflammatory syndrome in children (MIS-C) and adolescents associated with COVID-19: a meta-analysis. J Med Virol [Internet].

[30] Rodriguez-Smith JJ, Verweyen EL, Clay GM, Esteban YM, de Loizaga SR, Baker EJ (2021). Inflammatory biomarkers in COVID-19-associated multisystem inflammatory syndrome in children, Kawasaki disease, and macrophage activation syndrome: a cohort study. Lancet Rheumatol [Internet].

[31] Diaz F, Bustos BR, Yagnam F, Karsies TJ, Vásquez-Hoyos P, Jaramillo-Bustamante JC (2021). Comparison of interleukin-6 plasma concentration in multisystem inflammatory syndrome in children associated with SARS-CoV-2 and pediatric sepsis. Front Pediatr [Internet].

[32] Solé-Ribalta A, Bobillo-Pérez S, Jordan-García I (2022). A review of adrenomedullin in pediatric patients: a useful biomarker. Children [Internet].

[33] Tibi R, Hadash A, Khoury A, Butbul-Aviel Y, Ben-Ari J, Shavit I (2022). Emergency department levels of NT-proBNP and inotropic/vasoactive support in multi-inflammatory syndrome in children (MIS-C). Am J Emerg Med [Internet].

[34] Zhao Y, Patel J, Huang Y, Yin L, Tang L (2021). Cardiac markers of multisystem inflammatory syndrome in children (MIS-C) in COVID-19 patients: a meta-analysis. Am J Emerg Med [Internet].

[35] Toczyłowski K, Łasecka-Zadrożna J, Pałyga-Bysiecka I, Ludwikowska KM, Okarska-Napierała M, Dudek N (2022). Use of broad-spectrum antibiotics in children diagnosed with multisystem inflammatory syndrome temporarily associated with SARS-CoV-2 infection in Poland: the MOIS-CoR study. International Journal of Infectious Diseases [Internet].

[36] Yock-Corrales A, Lenzi J, Ulloa-Gutiérrez R, Gómez-Vargas J, Antúnez-Montes OY, Rios Aida JA (2021). High rates of antibiotic prescriptions in children with COVID-19 or multisystem inflammatory syndrome: a multinational experience in 990 cases from Latin America. Acta Paediatr [Internet].

